# Vitamin D and probiotic co-supplementation affects mental health, hormonal, inflammatory and oxidative stress parameters in women with polycystic ovary syndrome

**DOI:** 10.1186/s13048-019-0480-x

**Published:** 2019-01-21

**Authors:** Vahidreza Ostadmohammadi, Mehri Jamilian, Fereshteh Bahmani, Zatollah Asemi

**Affiliations:** 1 Research Center for Biochemistry and Nutrition in Metabolic Diseases, Kashan University of Medical Sciences, Kashan, Iran; 2 Endocrinology and Metabolism Research Center, Department of Gynecology and Obstetrics, School of Medicine, Arak University of Medical Sciences, Arak, Iran

**Keywords:** Vitamin D, Probiotic, Mental health, Hormonal profiles, Inflammatory markers, Polycystic ovary syndrome

## Abstract

**Objective:**

The aim of this study was to determine the effect of vitamin D and probiotic co-administration on mental health, hormonal, inflammatory and oxidative stress parameters in women with polycystic ovary syndrome (PCOS).

**Methods:**

This randomized, double-blinded, placebo-controlled clinical trial was carried out on 60 subjects, aged 18–40 years old. Subjects were randomly allocated to take either 50,000 IU vitamin D every 2 weeks plus 8 × 10^9^ CFU/day probiotic (*n* = 30) or placebo (n = 30) for 12 weeks.

**Results:**

Vitamin D and probiotic co-supplementation, compared with the placebo, significantly improved beck depression inventory [β (difference in the mean of outcomes measures between treatment groups) − 0.58; 95% CI, − 1.15, − 0.02; *P* = 0.04], general health questionnaire scores (β − 0.93; 95% CI, − 1.78, − 0.08; *P* = 0.03) and depression, anxiety and stress scale scores (β − 0.90; 95% CI, − 1.67, − 0.13; *P* = 0.02). Vitamin D and probiotic co-supplementation was associated with a significant reduction in total testosterone (β − 0.19 ng/mL; 95% CI, − 0.28, − 0.10; *P* < 0.001), hirsutism (β − 0.95; 95% CI, − 1.39, − 0.51; P < 0.001), high-sensitivity C-reactive protein (hs-CRP) (β − 0.67 mg/L; 95% CI, − 0.97, − 0.38; *P* < 0.001) and *malondialdehyde (MDA)* levels (β − 0.25 μmol/L; 95% CI, − 0.40, − 0.10; *P* = 0.001), and a significant increase in total antioxidant capacity (TAC) (β 82.81 mmol/L; 95% CI, 42.86, 122.75; P < 0.001) and total glutathione (GSH) levels (β 40.42 μmol/L; 95% CI, 4.69, 76.19; *P* = 0.02), compared with the placebo.

**Conclusions:**

Overall, the co-administration of vitamin D and probiotic for 12 weeks to women with PCOS had beneficial effects on mental health parameters, serum total testosterone, hirsutism, hs-CRP, plasma TAC, GSH and MDA levels.

**Trial Registration:**

This study was retrospectively registered in the Iranian website (www.irct.ir) for registration of clinical trials (IRCT20170513033941N37).

## Introduction

Polycystic ovarian syndrome (PCOS) is one of the most frequent gynecological endocrinopathy that occurs in premenopausal females [1]. Although the physiopathology of this syndrome is complicated and not yet completely elucidated; hyperandrogenism, inflammation and its permanent companions, and oxidative damage play central roles in PCOS [2, 3]. In fact, elevated androgen values may be due to the inflammatory response of the ovarian cells by free-radical species [4]. Furthermore, increased systemic inflammatory markers such as C-reactive protein (CRP) are related to the increased risk of type 2 diabetes mellitus (T2DM) and cardiovascular disease [5]. A recent meta-analysis documented that mental health disorders are common in patients with PCOS [6]. Also, hirsutism, menstrual irregularity, and acne impair the quality of life (QOL) in these women [7].

There is growing evidence suggesting the synergistic impact of combined vitamin D and probiotic administration on metabolic disorders, especially in patients with vitamin D deficiency, which might alleviate mental health parameters, and biomarkers of inflammation and oxidative stress in patients with metabolic syndrome and related disorders. The basis of this approach relies on probiotics effect increasing vitamin D levels [8]. In addition, probiotics might have synergistic effects with vitamin D, through improving the expression of vitamin D receptors [9]. Therefore, modulating the microbiota-gut-brain axis by probiotics plus improving vitamin D levels might provide a novel target to treat mental and metabolic disorders. Prior studies have documented that vitamin D deficiency (VDD) is prevalent among women with PCOS [10, 11]. Vitamin D deficiency is associated with elevated insulin resistance, and increased levels of total testosterone and dehydroepiandrosterone sulfate (DHEAS) in patients with PCOS [12]. Recent evidence showed that vitamin D at physiologic levels has a beneficial role on endometrial receptivity, whereas an excess of this molecule plays a detrimental role on oocytes development and embryo quality, probably due to its anti-estrogenic effect [13]. In addition, vitamin D was demonstrated to exert many physiological activities during the very early stages of gestation in perfect synchrony with progesterone [14]. In a meta-analysis, Akbari et al. [15] indicated that vitamin D administration to women with PCOS had beneficial impact on systemic inflammatory markers and oxidative damage. However, in another meta-analysis, vitamin D intake did not influence hormonal status in patients with PCOS [16]. Furthermore, taking 50,000 IU vitamin D for 12 weeks by patients with PCOS and VDD did not affect clinical status and hormonal profiles [17]. On the other hand, extensive evidence reveal that dysbiosis of gut microflora is involved in the pathogenesis of metabolic disturbances in PCOS [18]. Probiotics, as nonpathogenic micro-organisms, have shown promising effects on metabolic abnormalities such as increased inflammatory factors, oxidative stress, insulin resistance, and atherogenic dyslipidemia [19]. A 12-week trial using probiotic supplements in women with PCOS led to the amelioration of androgenic profiles, oxidative stress parameters and CRP concentrations [20].

Given the anti-inflammatory and antioxidant impacts of probiotic and vitamin D, we hypothesized that co-administration of both supplements might have synergistic effects on clinical status and biochemical parameters of women with PCOS. Therefore, we performed this trial to determine the impact of probiotic and vitamin D co-supplementation on hirsutism, mental health status, hormonal profiles, and biomarkers of inflammation and oxidative damage in patients with PCOS.

## Subjects and methods

### Participants

This randomized double-blinded, placebo-controlled trial was registered in the Iranian website for registration of clinical trials (http://www.irct.ir: IRCT20170513033941N37) and followed the Declaration of Helsinki and Good Clinical Practice guidelines. This investigation was carried out among 60 women with PCOS, diagnosed based on the Rotterdam criteria [21], with the body mass index (BMI) in the range of 17–34 kg/m^2^ and insulin resistance in the range of 1.4–4, aged 18–40 years old whom referred to the Naghavi Clinic in Kashan, Iran, between July and October 2018. The study was approved by the ethics committee of National Institute for Medical Research Development of Iran (NIMAD). Written informed consent was taken from all participants prior to the initiation of the trial. Exclusion criteria were as follows: pregnancy, lactation, adrenal hyperplasia, androgen-secreting tumors, hyperprolactinemia, thyroid dysfunction, and diabetes, women with psychological or psychiatric comorbidities such as anxiety or depressive symptoms at the enrollment.

### Supplementation

Subjects were randomized to take either 50,000 IU vitamin D every 2 weeks plus 8 × 10^9^ CFU/day probiotic (*n* = 30) or placebo (n = 30) for 12 weeks. Probiotic capsule contained four viable and freeze-dried strains: *Lactobacillus acidophilus*, *Bifidobacterium bifidum, Lactobacillus reuteri* and *Lactobacillus fermentum* (2 × 10^9^ CFU/g each). These are the basic minimal criteria which are considered in a high-quality probiotic supplement. Little is known about the ideal type and the dosage of probiotic used for patients with PCOS, so we selected the supplement and its dose based on previous published studies in diabetic patients with coronary heart disease [19]. Vitamin D, probiotic and placebos (corn oil and starch, respectively) were produced by Zahravi Pharmaceutical Company (Tabriz, Iran), LactoCare®, Zisttakhmir Company (Tehran, Iran) and Barij Essence Pharmaceutical Company (Kashan, Iran), respectively. They were completely identical in terms of their appearance, color, shape, size, smell, taste and packaging. Random assignment was conducted using computer-generated numbers. Randomization and allocation concealment were carried out for both the researchers and participants, by a trained staff at the gynecology clinic. The compliance rate was assessed by quantifying serum 25(OH) vitamin D levels. Intake of the probiotic, vitamin D3, and placebo capsules was monitored through asking participants to return the medication containers. To increase compliance rate, all patients received brief daily cell phone reminders to take the supplements. All subjects completed a 3-day diet recall at weeks 0, 4, 9 and 12 of the intervention. Daily macro- and micro-nutrient intakes were calculated using nutritionist IV software (First Databank, San Bruno, CA).

### Assessment of outcomes

Hormonal profiles were considered as the primary outcome. Mental health parameters, and biomarkers of inflammation and oxidative stress were recognized as the secondary outcomes.

### Clinical measures

Hirsutism was evaluated using a modified Ferriman-Gallwey (mFG) scoring system as 9 body areas including the upper lip, chin, chest, upper abdomen, lower abdomen, thighs, back, arm, and buttocks were investigating for hair; from 0 (no hair) to 4 (frankly virile) [22, 23]. Mental health was judged with beck depression inventory (BDI) [24], general health questionnaire-28 (GHQ-28) [25] and depression anxiety and stress scale (DASS) [26] at baseline and after the 12-week intervention. Quality of sleep was determined using PSQI [27].

### Biochemical assessment

Fasting blood samples (10 ml) were collected at baseline and the end of the intervention at Kashan reference laboratory. Serum total testosterone and sex hormone-binding globulin (SHBG) with inter- and intra-assay with inter- and intra-assay CVs below 7% were quantified using ELISA kits (DiaMetra, Milano, Italy). Serum 25-hydroxyvitamin D concentrations were determined using an ELISA kit (IDS, Boldon, UK) and enzyme-linked immunosorbent assay with inter- and intra-assay CVs below 7%. Serum high sensitivity C-reactive protein (hs-CRP) concentrations were measured using an ELISA kit (LDN, Nordhorn, Germany) with inter- and intra-assay CVs below 7%. The plasma NO levels were measured using Griess method [28], total antioxidant capacity (TAC) concentrations using Benzie and Strain method [29], total glutathione (GSH) using Beutler method [30] and malondialdehyde (MDA) concentrations thiobarbituric acid reactive substances spectrophotometric test [31] with CVs below 5%.

### Sample size

We used a randomized clinical trial sample size calculation formula where type one (α) and type two errors (beta) were 0.05, and 0.20 (power = 80%), respectively. According to a previous published study [32], we used 0.48 ng/mL as the difference in mean (d) and 0.60 ng/mL as SD for total testosterone as the key variable. Using the formula, we needed 25 participants in each group; after allowing for 5 dropouts in each group, the final sample size was 30 persons in each group. The standardized effect size was equal to 0.48/0.60 = 0.8 which is considered as a large effect size according to Cohen [33]. Using SD = 0.60, we had at least 80% power (probability) of detecting a difference equal to or greater than 0.48 (if it really exists) as statistically significant at the 5% level.

### Statistical analyses

The Kolmogorov-Smirnov test was conducted to determine the normality of data. Differences in anthropometric measurements and dietary intakes between treatment groups were determined using independent-sample *t*-tests. Multiple linear regression models were used to assess the treatment effects on study outcomes, after adjusting for confounding parameters, including age and BMI. The effect sizes (β) were presented as the mean differences with 95% confidence intervals between two groups. *P*-values < 0.05 were considered statistically significant. All statistical analyses were done using the Statistical Package for Social Science version 18 (SPSS Inc., Chicago, Illinois, USA).

## Results

**Fig. 1:**
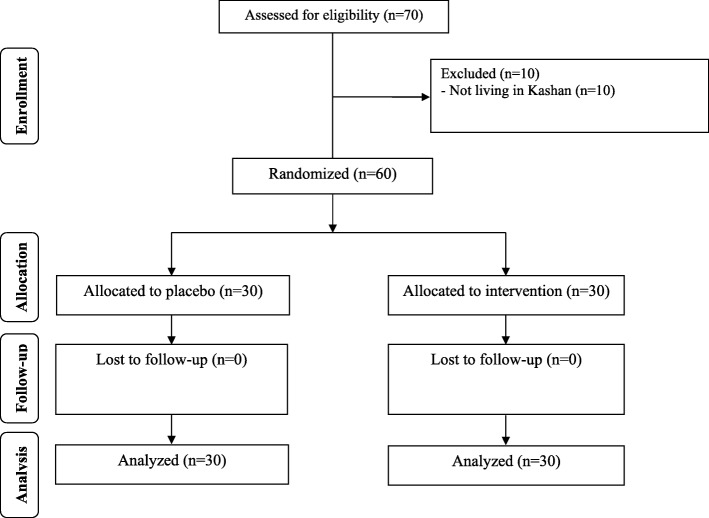
Summary of patient flow diagram

As demonstrated in the study flow diagram **(**Fig. 1**),** 60 participants [placebo (*n* = 30) and vitamin D plus probiotic supplements (n = 30)] completed the trial. No side effects were reported following co-administration of vitamin D and probiotic capsules in patients with PCOS throughout the study.

Mean age, height, baseline and end-of-trial weight and BMI of study participants were not statistically different between both groups (Table 1). There was no statistically significant difference in terms of dietary macro- and micro-nutrient intakes between vitamin D plus probiotic, and placebo groups (Data not shown).

**Table 1:** General characteristics of study participants^1^

	Placebo group (*n* = 30)	Vitamin D plus probiotic group (*n* = 30)	P^2^
Age (y)	25.4 ± 5.1	24.4 ± 4.7	0.40
Height (cm)	162.9 ± 6.8	163.0 ± 8.5	0.97
Weight at study baseline (kg)	66.3 ± 11.4	64.8 ± 13.2	0.63
Weight at end-of-trial (kg)	65.8 ± 11.1	64.1 ± 13.0	0.57
Weight change (kg)	-0.5 ± 1.2	−0.7 ± 0.5	0.31
BMI at study baseline (kg/m^2^)	25.1 ± 4.9	24.3 ± 4.2	0.51
BMI at end-of-trial (kg/m^2^)	24.9 ± 4.8	24.1 ± 4.1	0.46
BMI change (kg/m^2^)	−0.2 ± 0.5	− 0.3 ± 0.2	0.35

^1^Data are means± SDs

^2^Obtained from independent *t*-test

After the 12-week intervention, vitamin D and probiotic co-supplementation significantly improved BDI [β (difference in the mean of outcomes measures between treatment groups) -0.58; 95% CI, − 1.15, − 0.02; *P* = 0.04], GHQ (β − 0.93; 95% CI, − 1.78, − 0.08; *P* = 0.03) and DASS (β − 0.90; 95% CI, − 1.67, − 0.13; *P* = 0.02), compared with the placebo **(**Table 2). Vitamin D and probiotic co-supplementation was associated with a significant reduction in total testosterone (β − 0.19 ng/mL; 95% CI, − 0.28, − 0.10; *P* < 0.001), hirsutism (β − 0.95; 95% CI, − 1.39, − 0.51; *P* < 0.001), hs-CRP (β − 0.67 mg/L; 95% CI, − 0.97, − 0.38; *P* < 0.001) and *MDA* levels (β − 0.25 μmol/L; 95% CI, − 0.40, − 0.10; *P* = 0.001), and a significant increase in TAC (β 82.81 mmol/L; 95% CI, 42.86, 122.75; P < 0.001) and GSH levels (β 40.42 μmol/L; 95% CI, 4.69, 76.19; P = 0.02) compared with the placebo. Co-supplementation did not affect serum SHBG and plasma NO levels, as well as acne and alopecia.

**Table 2:** Mental health parameters and metabolic profiles at baseline and after the 12-week intervention in women with polycystic ovary syndrome that received either vitamin D plus probiotic supplements or placebo

Variables	Placebo group (*n* = 30)		Vitamin D plus probiotic group (*n* = 30)		Difference in outcome measures between vitamin D plus probiotic and placebo groups^1^	
	Baseline	Week 12	Baseline	Week 12	β (95% CI)	P^2^
25-hydroxyvitamin D (ng/mL)	12.9 ± 3.2	13.3 ± 2.7	11.1 ± 4.1	24.4 ± 5.6	12.69 (11.09, 14.29)	< 0.001
BDI total scores	13.8 ± 3.6	13.2 ± 3.7	12.9 ± 4.1	11.9 ± 3.4	−0.58 (−1.15, − 0.02)	0.04
GHQ scores	42.7 ± 9.1	41.9 ± 8.9	40.4 ± 6.7	38.7 ± 6.8	−0.93 (−1.78, − 0.08)	0.03
DASS scores	82.5 ± 11.8	80.8 ± 12.4	81.5 ± 12.2	78.9 ± 12.3	−0.90 (−1.67, − 0.13)	0.02
PSQI	7.5 ± 2.8	6.5 ± 3.0	7.8 ± 2.7	5.8 ± 2.1	−0.73 (−2.11,-0.63)	0.28
Total testosterone (ng/mL)	1.1 ± 0.4	1.1 ± 0.3	1.0 ± 0.3	0.9 ± 0.2	−0.19 (− 0.28, − 0.10)	< 0.001
SHBG (nmol/L)	40.2 ± 5.0	40.3 ± 5.3	38.4 ± 5.9	39.6 ± 5.7	0.92 (−0.45, 2.31)	0.18
mF-G scores	13.9 ± 3.0	13.6 ± 3.0	14.98 ± 3.2	13.5 ± 3.3	−0.95 (−1.39, − 0.51)	< 0.001
hs-CRP (mg/L)	3.7 ± 1.0	3.8 ± 1.3	3.8 ± 1.1	3.2 ± 0.8	−0.67 (− 0.97, − 0.38)	< 0.001
NO (μmol/L)	33.3 ± 4.9	32.3 ± 5.5	34.2 ± 1.0	33.6 ± 0.8	0.37 (−0.48, 1.23)	0.38
TAC (mmol/L)	835.1 ± 101.7	830.9 ± 93.8	850.5 ± 82.7	919.2 ± 85.4	82.81 (42.86, 122.75)	< 0.001
GSH (μmol/L)	475.8 ± 71.7	491.7 ± 81.0	510.4 ± 99.3	556.5 ± 97.2	40.42 (4.69, 76.15)	0.02
MDA (μmol/L)	2.7 ± 0.5	2.6 ± 0.5	2.9 ± 0.3	2.5 ± 0.2	−0.25 (−0.40, −0.10)	0.001

Data are mean ± SDs

^1^“Outcome measures” refers to the change in values of measures of interest between baseline and week 12. β [difference in the mean outcomes measures between treatment groups (vitamin D plus probiotic group = 1 and placebo group = 0)]

^2^Obtained from multiple regression model (adjusted for baseline values of each biochemical variables, age and baseline BMI)

BDI, beck depression inventory; DASS, depression anxiety and stress scale; GHQ, general health questionnaire; GSH, total glutathione; hs-CRP, high-sensitivity C-reactive protein; mF-G, modified Ferriman Gallwey; MDA, malondialdehyde; NO, nitric oxide; PSQI, Pittsburgh Sleep Quality Index; SHBG, sex hormone-binding globulin; TAC, total antioxidant capacity

## Discussion

In the current study, we investigated the effects of vitamin D and probiotic co-supplementation for 12 weeks on mental health, hormonal, inflammatory and oxidative stress parameters among women with PCOS. We found that the co-administration of vitamin D and probiotic for 12 weeks to women with PCOS had beneficial effects on mental health parameters, serum total testosterone, hirsutism, hs-CRP, plasma TAC, GSH and MDA levels, but did not affect serum SHBG, plasma NO levels, acne and alopecia.

### Effects on mental health

We found vitamin D and probiotic co-administration for 12 weeks to women with PCOS significantly reduced BDI, GHQ and DASS scores, yet did not influence PSQI index. Prior reports have documented the association between mood disorders and gastrointestinal microbiota, indicating the role of the gut-brain axis in the physiopathology of clinical depression [34]. Moreover, microflora biosynthesis and the regulation of neurotransmitters, including GABA [35] and serotonin [36] are probable mechanisms that gut bacteria can affect mental status. In a recent meta-analysis, probiotic consumption did not affect depressive symptoms in healthy people [37]. In addition, Ju et al. [38] indicated an inverse relationship between serum vitamin D values and depression on a pooled meta-analysis of cross-sectional and cohort studies. Vitamin D contributes to various brain processes such as neuroprotection, neuroimmunomodulation, and brain development, suggesting that mental health disorders may be correlated with VDD [39, 40]. Vitamin D may ameliorate mental health disorders, via up-regulation of tyrosine hydroxylase gene expression and augmentation of the bioavailability of various neurotransmitters, including norepinephrine and dopamine [41]. Furthermore, vitamin D intake had an insignificant impact on depression in adults [42]. On the other hand, taking oral preparations of isoflavones (40 mg), calcium (500 mg) vitamin D (300 IU) and inulin (3 g) for 12 months by menopausal women significantly improved quality of life, sexual function, body composition and metabolic parameters [43]. Unlike, Raygan et al. [44] demonstrated that consumption of 50,000 IU/biweekly cholecalciferol plus one probiotic capsule daily by type 2 diabetic patients with ischemic heart disease significantly improved depression and anxiety indices. The synergism between the immunomodulatory, antioxidant, and anti-inflammatory properties of both supplements might enhance their impact on mental health parameters.

### Effects on hirsutism and hormonal profiles

Our results provided evidence that vitamin D and probiotic co-supplementation for 12 weeks in women with PCOS significantly improved hirsutism and total testosterone concentrations, but did not affect SHBG values. To date, little is known about drug metabolism in women with PCOS. This important gap in the literature could have significant implications for therapeutic approaches and future perspectives: first, the dosage of drugs commonly used for the treatment of women with PCOS should be tailored according to each patient’s characteristics; second, implementing new clinical trials in order to identify the best pharmacologic strategy for patients with PCOS undergoing in vitro fertilization (IVF); finally, advising to create an international expert panel to investigate the drug metabolism in women with PCOS [45]. Cumulative evidence from IVF studies has proposed that fertilization rate decreases significantly with increasing levels of 25OH-D in follicular fluid; in addition, vitamin D concentrations in the follicular fluid are negatively correlated with the quality of embryos and the higher values of vitamin D are associated with lower possibility to achieve pregnancy [13]. In addition, vitamin D is involved in the modulation of the reproductive process in women due to the expression of VDR and 1α-hydroxylase in reproductive tissues, including ovary, uterus, placenta, pituitary and hypothalamus [46]. Combined therapy with vitamin D and probiotic may have the positive effects on outcome of assisted reproductive technologies. Earlier, it was reported that taking myo-inositol plus melatonin showed an improved number of good quality oocytes and embryos, with reduced follicle stimulating hormone levels and days of treatment during cycles IVF [47]. Maktabi et al. [17] showed that the intake of cholecalciferol (50,000 IU/biweekly for 12 weeks) by patients with PCOS did not influence hirsutism, total testosterone, and SHBG levels. Furthermore, taking high-dose vitamin D3 (12,000 IU/day cholecalciferol) for 3 months by patients with PCOS did not affect androgen profiles (total- and free testosterone levels) [48]. In a meta-analysis, probiotic supplementation had no significant effect on DHEAS levels [49].

Hyperandrogenism, a hallmark of PCOS, contributed to clinical features of this syndrome such as acne, hirsutism, menstrual disturbances, and anovulation [50]. It has been shown that reduction in androgen concentrations is correlated with the amelioration in ovulatory functions, decreasing hirsutism, and improving QOL [51, 52]. Probiotic may improve androgenic profiles via elevating insulin sensitivity, enhancing absorption and digestion of dietary nutrients, modulating gut microflora and gut-brain axis [53, 54]. Impact of vitamin D on activity and expression of various enzymes related to the steroidogenesis pathway may explain the decrement in circulating total testosterone concentrations [55]. We hypothesized that combination therapy with vitamin D and probiotic in patients with PCOS may work better than a single supplementation alone. Combined vitamin D and probiotic supplementation might also have a strong synergistic effect on hormonal profiles and biomarkers of inflammation and oxidative stress. In a study by Jamilian et al. [56], it was seen that vitamin D and probiotic co-supplementation in women with gestational diabetes had beneficial effects on metabolic status compared with probiotic alone. Furthermore, probiotics might have synergistic effects with vitamin D through improving the expression of vitamin D receptors [9]. The majority of subjects in this study had vitamin D deficiency, so decreased inflammation and oxidative stress by vitamin D and probiotic may improve hormonal profiles. To our best knowledge, data on the effects of vitamin D plus probiotic supplementation, compared with only vitamin D or probiotic, on hormonal profiles, and biomarkers of inflammation and oxidative stress are limited. Therefore, further studies are required with single supplementation of each compared with co-supplementation to assess the beneficial effects on metabolic profiles.

### Effects on biomarkers of inflammation and oxidative stress

Our results revealed that combined vitamin D and probiotic administration to women with PCOS led to a significant reduction in serum hs-CRP and plasma MDA levels, and a significant elevation in plasma GSH and TAC levels after 12 weeks. Based on current evidence, vitamin D and probiotics may have beneficial impact on inflammation and oxidative damage. Razzaghi et al. [57] indicated that vitamin D supplementation for 12 weeks to patients with diabetic foot ulcer had beneficial effects on hs-CRP and MDA levels, but did not affect TAC and GSH levels. In a recent meta-analysis of RCTs, Mansournia et al. [58] showed that vitamin D intake improved markers of oxidative damage and inflammation in diabetic people. In another meta-analysis, vitamin D intake caused a significant reduction in hs-CRP values [59]. On the other hand, the consumption of probiotic supplements for 12-week by women with PCOS decreased CRP and MDA levels [20]. In addition, taking probiotic for 12 weeks by patients with multiple sclerosis had favorable influences on a few systemic inflammatory markers and oxidative stress [60]. However, in a meta-analysis performed among subjects with T2DM, probiotic use did not affect CRP concentrations [61]. Also, vitamin D consumption had no significant impact on inflammatory biomarkers in overweight and obese people [62]. Previous published reports demonstrated controversial findings regarding the impact of vitamin D and probiotic supplementation on markers of inflammation and oxidative damage. This may be due to the variations in study conditions, different dosages of supplements, and differences in intervention period. Increased oxidative damage and inflammatory cytokines are related to increased risk of hyperandrogenism, insulin resistance, cardiovascular events, and diabetes in PCOS [63, 64]. Probiotic consumption may reduce inflammatory cytokines, lipid peroxidation, and oxidative damage via producing short chain fatty acid in the intestine and reduction in generation of hydrogen peroxide radicals [65]. Furthermore, vitamin D can suppress nuclear transcription factor kappa-B and decrease the production of free radicals and pro-inflammatory cytokines [66].

The present trial has few limitations. We did not determine the loads of fecal bacteria and microbiome characterization before, during, and after intervention. Moreover, we did not investigate the impact of vitamin D and probiotic co-administration on other markers of oxidative damage and inflammation.

## Conclusions

Overall, the co-administration of vitamin D and probiotic for 12 weeks to women with PCOS had beneficial effects on mental health parameters, serum total testosterone, hirsutism, hs-CRP, plasma TAC, GSH and MDA levels, but did not affect serum SHBG, plasma NO levels, acne and alopecia.

## Abbreviations

GSH: total glutathione
hs-CRP: high-sensitivity C-reactive protein
MDA: malondialdehyde
mF-G: modified Ferriman Gallwey
NO: nitric oxide
PCOS: polycystic ovary syndrome
SHBG: sex hormone-binding globulin
TAC: total antioxidant capacity

## References

[1] Huang CC, Tien YJ, Chen MJ, Chen CH, Ho HN, Yang YS (2015). Symptom patterns and phenotypic subgrouping of women with polycystic ovary syndrome: association between endocrine characteristics and metabolic aberrations. Hum Reprod.

[2] Artimani T, Karimi J, Mehdizadeh M, Yavangi M, Khanlarzadeh E, Ghorbani M (2018). Evaluation of pro-oxidant-antioxidant balance (PAB) and its association with inflammatory cytokines in polycystic ovary syndrome (PCOS). Gynecol Endocrinol.

[3] Walters KA, Gilchrist RB, Ledger WL, Teede HJ, Handelsman DJ, Campbell RE (2018). New perspectives on the pathogenesis of PCOS: neuroendocrine origins. Trends Endocrinol Metab.

[4] Gonzalez F, Rote NS, Minium J, Kirwan JP (2006). Reactive oxygen species-induced oxidative stress in the development of insulin resistance and hyperandrogenism in polycystic ovary syndrome. J Clin Endocrinol Metab.

[5] Kelly CC, Lyall H, Petrie JR, Gould GW, Connell JM, Sattar N (2001). Low grade chronic inflammation in women with polycystic ovarian syndrome. J Clin Endocrinol Metab.

[6] Cooney LG, Lee I, Sammel MD, Dokras A (2017). High prevalence of moderate and severe depressive and anxiety symptoms in polycystic ovary syndrome: a systematic review and meta-analysis. Hum Reprod.

[7] van Zuuren EJ, Fedorowicz Z (2016). Interventions for hirsutism excluding laser and photoepilation therapy alone: abridged Cochrane systematic review including GRADE assessments. Br J Dermatol.

[8] Jones ML, Martoni CJ, Prakash S (2013). Oral supplementation with probiotic L. reuteri NCIMB 30242 increases mean circulating 25-hydroxyvitamin D: a post hoc analysis of a randomized controlled trial. J Clin Endocrinol Metab.

[9] Shang M, Sun J (2017). Vitamin D/VDR, probiotics, and gastrointestinal diseases. Curr Med Chem.

[10] Thomson RL, Spedding S, Brinkworth GD, Noakes M, Buckley JD (2013). Seasonal effects on vitamin D status influence outcomes of lifestyle intervention in overweight and obese women with polycystic ovary syndrome. Fertil Steril.

[11] He C, Lin Z, Robb SW, Ezeamama AE (2015). Serum vitamin D levels and polycystic ovary syndrome: a systematic review and meta-analysis. Nutrients.

[12] Hahn S, Haselhorst U, Tan S, Quadbeck B, Schmidt M, Roesler S (2006). Low serum 25-hydroxyvitamin D concentrations are associated with insulin resistance and obesity in women with polycystic ovary syndrome. Exp Clin Endocrinol Diabetes.

[13] Lagana AS, Vitale SG, Ban Frangez H, Vrtacnik-Bokal E, D'Anna R (2017). Vitamin D in human reproduction: the more, the better? An evidence-based critical appraisal. Eur Rev Med Pharmacol Sci.

[14] Monastra G, De Grazia S, De Luca L, Vittorio S, Unfer V, Vitamin D (2018). A steroid hormone with progesterone-like activity. Eur Rev Med Pharmacol Sci.

[15] Akbari M, Ostadmohammadi V, Lankarani KB, Tabrizi R, Kolahdooz F, Heydari ST (2018). The effects of vitamin D supplementation on biomarkers of inflammation and oxidative stress among women with polycystic ovary syndrome: a systematic review and meta-analysis of randomized controlled trials. Horm Metab Res.

[16] Xue Y, Xu P, Xue K, Duan X, Cao J, Luan T (2017). Effect of vitamin D on biochemical parameters in polycystic ovary syndrome women: a meta-analysis. Arch Gynecol Obstet.

[17] Maktabi M, Chamani M, Asemi Z (2017). The effects of vitamin D supplementation on metabolic status of patients with polycystic ovary syndrome: a randomized, double-blind, placebo-controlled trial. Horm Metab Res.

[18] Lindheim L, Bashir M, Munzker J, Trummer C, Zachhuber V, Leber B (2017). Alterations in gut microbiome composition and barrier function are associated with reproductive and metabolic defects in women with polycystic ovary syndrome (PCOS): a pilot study. PLoS One.

[19] Raygan F, Ostadmohammadi V, Asemi Z. The effects of probiotic and selenium co-supplementation on mental health parameters and metabolic profiles in type 2 diabetic patients with coronary heart disease: a randomized, double-blind, placebo-controlled trial. Clin Nutr. 2018. 10.1016/j.clnu.2018.07.017. 10.1016/j.clnu.2018.07.017 30057015

[20] Karamali M, Eghbalpour S, Rajabi S, Jamilian M, Bahmani F, Tajabadi-Ebrahimi M (2018). Effects of probiotic supplementation on hormonal profiles, biomarkers of inflammation and oxidative stress in women with polycystic ovary syndrome: a randomized, double-blind, placebo-controlled trial. Arch Iran Med.

[21] Rotterdam ESHRE (2004). ASRM-sponsored PCOS consensus workshop group. Revised 2003 consensus on diagnostic criteria and long-term health risks related to polycystic ovary syndrome. Fertil Steril.

[22] Hatch R, Rosenfield RL, Kim MH, Tredway D (1981). Hirsutism: implications, etiology, and management. Am J Obstet Gynecol.

[23] Ramezani Tehrani F, Minooee S, Azizi F (2014). Validation of a simplified method to assess hirsutism in the Iranian population. Eur J Obstet Gynecol Reprod Biol.

[24] Beck AT, Ward CH, Mendelson M, Mock J, Erbaugh J (1961). An inventory for measuring depression. Arch Gen Psychiatry.

[25] Goldberg DP, Hillier VF (1979). A scaled version of the general health questionnaire. Psychol Med.

[26] Crawford JR, Henry JD (2004). The positive and negative affect schedule (PANAS): construct validity, measurement properties and normative data in a large non-clinical sample. Br J Clin Psychol.

[27] Buysse DJ, Reynolds CF, Monk TH, Berman SR, Kupfer DJ (1989). The Pittsburgh sleep quality index: a new instrument for psychiatric practice and research. Psychiatry Res.

[28] Tatsch E, Bochi GV, Pereira Rda S, Kober H, Agertt VA, de Campos MM (2011). A simple and inexpensive automated technique for measurement of serum nitrite/nitrate. Clin Biochem.

[29] Benzie IF, Strain JJ (1996). The ferric reducing ability of plasma (FRAP) as a measure of "antioxidant power": the FRAP assay. Anal Biochem.

[30] Beutler E, Gelbart T (1985). Plasma glutathione in health and in patients with malignant disease. J Lab Clin Med.

[31] Janero DR (1990). Malondialdehyde and thiobarbituric acid-reactivity as diagnostic indices of lipid peroxidation and peroxidative tissue injury. Free Radic Biol Med.

[32] Jamilian M, Foroozanfard F, Rahmani E, Talebi M, Bahmani F, Asemi Z. Effect of two different doses of vitamin D supplementation on metabolic profiles of insulin-resistant patients with polycystic ovary syndrome. Nutrients. 2017;9. 10.3390/nu9121280. 10.3390/nu9121280 PMC5748731 29186759

[33] Mansournia MA, Altman DG (2018). Invited commentary: methodological issues in the design and analysis of randomised trials. Br J Sports Med.

[34] Schmidt C (2015). Mental health: thinking from the gut. Nature.

[35] Barrett E, Ross RP, O'Toole PW, Fitzgerald GF, Stanton C (2012). Gamma-aminobutyric acid production by culturable bacteria from the human intestine. J Appl Microbiol.

[36] Yano JM, Yu K, Donaldson GP, Shastri GG, Ann P, Ma L (2015). Indigenous bacteria from the gut microbiota regulate host serotonin biosynthesis. Cell.

[37] Ng QX, Peters C, Ho CYX, Lim DY, Yeo WS (2018). A meta-analysis of the use of probiotics to alleviate depressive symptoms. J Affect Disord.

[38] Ju SY, Lee YJ, Jeong SN (2013). Serum 25-hydroxyvitamin D levels and the risk of depression: a systematic review and meta-analysis. J Nutr Health Aging.

[39] Fernandes de Abreu DA, Eyles D, Feron F, Vitamin D (2009). A neuro-immunomodulator: implications for neurodegenerative and autoimmune diseases. Psychoneuroendocrinology.

[40] Bertone-Johnson ER (2009). Vitamin D and the occurrence of depression: causal association or circumstantial evidence?. Nutr Rev.

[41] Humble MB (2010). Vitamin D, light and mental health. J Photochem Photobiol B.

[42] Gowda U, Mutowo MP, Smith BJ, Wluka AE, Renzaho AM (2015). Vitamin D supplementation to reduce depression in adults: meta-analysis of randomized controlled trials. Nutrition.

[43] Vitale SG, Caruso S, Rapisarda AMC, Cianci S, Cianci A (2018). Isoflavones, calcium, vitamin D and inulin improve quality of life, sexual function, body composition and metabolic parameters in menopausal women: result from a prospective, randomized, placebo-controlled, parallel-group study. Prz Menopauzalny.

[44] Raygan F, Ostadmohammadi V, Bahmani F, Asemi Z (2018). The effects of vitamin D and probiotic co-supplementation on mental health parameters and metabolic status in type 2 diabetic patients with coronary heart disease: a randomized, double-blind, placebo-controlled trial. Prog Neuro-Psychopharmacol Biol Psychiatry.

[45] Reyes-Munoz E, Sathyapalan T, Rossetti P, Shah M, Long M, Buscema M (2018). Polycystic ovary syndrome: implication for drug metabolism on assisted reproductive techniques-a literature review. Adv Ther.

[46] Muscogiuri G, Altieri B, de Angelis C, Palomba S, Pivonello R, Colao A (2017). Shedding new light on female fertility: the role of vitamin D. Rev Endocr Metab Disord.

[47] Vitale SG, Rossetti P, Corrado F, Rapisarda AM, La Vignera S, Condorelli RA (2016). How to achieve high-quality oocytes? The key role of myo-inositol and melatonin. Int J Endocrinol.

[48] Raja-Khan N, Shah J, Stetter CM, Lott ME, Kunselman AR, Dodson WC (2014). High-dose vitamin D supplementation and measures of insulin sensitivity in polycystic ovary syndrome: a randomized, controlled pilot trial. Fertil Steril.

[49] Liao D, Zhong C, Li C, Mo L, Liu Y. Meta-analysis of the effects of probiotic supplementation on glycaemia, lipidic profiles, weight loss and C-reactive protein in women with polycystic ovarian syndrome. Minerva Med. 2018. 10.23736/S0026-4806.18.05728-2 30256077

[50] Dumitrescu R, Mehedintu C, Briceag I, Purcarea VL, Hudita D (2015). The polycystic ovary syndrome: an update on metabolic and hormonal mechanisms. J Med Life.

[51] Dokras A, Sarwer DB, Allison KC, Milman L, Kris-Etherton PM, Kunselman AR (2016). Weight loss and lowering androgens predict improvements in health-related quality of life in women with PCOS. J Clin Endocrinol Metab.

[52] Boztosun A, Acmaz G, Ozturk A, Muderris II (2013). Clinical efficacy of low dose flutamide plus Diane-35 in the treatment of idiopathic hirsutism and polycystic ovary syndrome. Ginekol Pol.

[53] Crommen S, Simon MC. Microbial regulation of glucose metabolism and insulin resistance. Genes. 2017;9. 10.3390/genes9010010 PMC5793163 29286343

[54] Saydam BO, Yildiz BO (2016). Gut-brain axis and metabolism in polycystic ovary syndrome. Curr Pharm Des.

[55] Pal L, Berry A, Coraluzzi L, Kustan E, Danton C, Shaw J (2012). Therapeutic implications of vitamin D and calcium in overweight women with polycystic ovary syndrome. Gynecol Endocrinol.

[56] Jamilian M, Amirani E, Asemi Z. The effects of vitamin D and probiotic co-supplementation on glucose homeostasis, inflammation, oxidative stress and pregnancy outcomes in gestational diabetes: a randomized, double-blind, placebo-controlled trial. Clin Nutr. 2018. 10.1016/j.clnu.2018.10.028. 10.1016/j.clnu.2018.10.028 30459099

[57] Razzaghi R, Pourbagheri H, Momen-Heravi M, Bahmani F, Shadi J, Soleimani Z (2017). The effects of vitamin D supplementation on wound healing and metabolic status in patients with diabetic foot ulcer: a randomized, double-blind, placebo-controlled trial. J Diabetes Complicat.

[58] Mansournia MA, Ostadmohammadi V, Doosti-Irani A, Ghayour-Mobarhan M, Ferns G, Akbari H (2018). The effects of vitamin D supplementation on biomarkers of inflammation and oxidative stress in diabetic patients: a systematic review and meta-analysis of randomized controlled trials. Horm Metab Res.

[59] Chen N, Wan Z, Han SF, Li BY, Zhang ZL, Qin LQ (2014). Effect of vitamin D supplementation on the level of circulating high-sensitivity C-reactive protein: a meta-analysis of randomized controlled trials. Nutrients.

[60] Kouchaki E, Tamtaji OR, Salami M, Bahmani F, Daneshvar Kakhaki R, Akbari E (2017). Clinical and metabolic response to probiotic supplementation in patients with multiple sclerosis: a randomized, double-blind, placebo-controlled trial. Clin Nutr.

[61] Kasinska MA, Drzewoski J (2015). Effectiveness of probiotics in type 2 diabetes: a meta-analysis. Pol Arch Med Wewn.

[62] Jamka M, Wozniewicz M, Walkowiak J, Bogdanski P, Jeszka J, Stelmach-Mardas M (2016). The effect of vitamin D supplementation on selected inflammatory biomarkers in obese and overweight subjects: a systematic review with meta-analysis. Eur J Nutr.

[63] Pawelczak M, Rosenthal J, Milla S, Liu YH, Shah B (2014). Evaluation of the pro-inflammatory cytokine tumor necrosis factor-alpha in adolescents with polycystic ovary syndrome. J Pediatr Adolesc Gynecol.

[64] Boots CE, Jungheim ES (2015). Inflammation and human ovarian follicular dynamics. Semin Reprod Med.

[65] Sadrzadeh-Yeganeh H, Elmadfa I, Djazayery A, Jalali M, Heshmat R, Chamary M (2010). The effects of probiotic and conventional yoghurt on lipid profile in women. Br J Nutr.

[66] Al-Rasheed NM, Al-Rasheed NM, Bassiouni YA, Hasan IH, Al-Amin MA, Al-Ajmi HN (2015). Vitamin D attenuates pro-inflammatory TNF-alpha cytokine expression by inhibiting NF-small ka, CyrillicB/p65 signaling in hypertrophied rat hearts. J Physiol Biochem.

